# Changes in Population Age-Structure Obscure the Temperature-Size Rule in Marine Cyanobacteria

**DOI:** 10.3389/fmicb.2020.02059

**Published:** 2020-08-28

**Authors:** Antonio S. Palacio, Ana María Cabello, Francisca C. García, Abbrar Labban, Xosé Anxelu G. Morán, Laurence Garczarek, Laura Alonso-Sáez, Ángel López-Urrutia

**Affiliations:** ^1^AZTI, Marine Research, Basque Research and Technology Alliance (BRTA), Sukarrieta, Spain; ^2^Spanish Institute of Oceanography (IEO), Oceanographic Center of Malaga, Málaga, Spain; ^3^Environment and Sustainability Institute, University of Exeter, Exeter, United Kingdom; ^4^King Abdullah University of Science and Technology, Red Sea Research Center, Thuwal, Saudi Arabia; ^5^Spanish Institute of Oceanography (IEO), Oceanographic Center of Gijón/Xixón, Gijón, Spain; ^6^Sorbonne Université, CNRS, UMR 7144 Adaptation and Diversity in the Marine Environment (AD2M), Roscoff, France

**Keywords:** temperature-size rule, *Prochlorococcus*, *Synechococcus*, cell size, temperature, cell division, cell cycle

## Abstract

The temperature-size Rule (TSR) states that there is a negative relationship between ambient temperature and body size. This rule has been independently evaluated for different phases of the life cycle in multicellular eukaryotes, but mostly for the average population in unicellular organisms. We acclimated two model marine cyanobacterial strains (*Prochlorococcus marinus* MIT9301 and *Synechococcus sp.* RS9907) to a gradient of temperatures and measured the changes in population age-structure and cell size along their division cycle. Both strains displayed temperature-dependent diel changes in cell size, and as a result, the relationship between temperature and average cell size varied along the day. We computed the mean cell size of new-born cells in order to test the prediction of the TSR on a single-growth stage. Our work reconciles previous inconsistent results when testing the TSR on unicellular organisms, and shows that when a single-growth stage is considered the predicted negative response to temperature is revealed.

## Introduction

Organism size is one of the most fundamental functional traits in ecology affecting all levels of biological organization from individual fitness to ecosystem processes (Brown et al., 2004; Kingsolver and Huey, 2008). Marine microbial communities are no exception (Andersen et al., 2016). For example, phytoplankton cell size determines resource utilization, primary production and downward export, hence affecting biogeochemical cycling in the oceans (Chisholm, 1992). Organism size can respond to different environmental conditions, with temperature being one of the main regulating factors (Kingsolver and Huey, 2008). An inverse correlation between body size and ambient temperature exists in a wide range of organisms, including bacteria, protist, plants and animals (Forster et al., 2012). For individual species, the “Temperature size rule” (TSR) (Atkinson, 1994) considers this inverse correlation between temperature and body size as the result of phenotypic plasticity. Several studies have found support for the TSR analyzing different types of organisms. However, the relationship between temperature and body size has seldom been explored in environmentally relevant microorganisms such as marine phytoplankton, even if temperature has a key role in regulating their global distribution (Flombaum et al., 2013; Sunagawa et al., 2015). A meta-analysis carried out on aquatic autotrophic and heterotrophic protists found support for the TSR, with cell volume decreasing by 2.5% for each increment of 1°C (Atkinson et al., 2003). Yet, individual responses to temperature were variable, and these authors failed to find a significant negative linear relationship in 24 out of 44 datasets examined, highlighting the lack of universality of this rule.

The cyanobacteria *Prochlorococcus marinus* and *Synechococcus* sp. are key members of phytoplankton communities (Campbell et al., 1994; Li and Url, 1994), and responsible for a major share of the global marine productivity (Iturriaga and Marra, 1988; Burkill et al., 1993; Vaulot et al., 1995; Liu et al., 1997; Flombaum et al., 2013). Previous studies exploring the relationship between temperature and cell size in *Prochlorococcus* and *Synechococcus* found contradictory results, both in natural communities and in culture. Morán et al. (2010) found a negative trend between temperature and mean cell size in *Prochlorococcus* and *Synechococcus* NE Atlantic populations, while Sato et al. (2015) did not find any significant relationship in the Pacific Ocean. In the Indian Ocean, a decrease of cell size with depth was reported, which was attributed to the combined effects of light-limitation and low temperature (Wei et al., 2018).

Besides these field and community-level experimental studies, some experiments with single strains have also measured the degree of plastic response of cell size to temperature (i.e., the TSR). The few studies that have measured this parameter on *Prochlorococcus* cultures acclimated to different temperatures (Fu et al., 2007; Kulk et al., 2012; Martiny et al., 2016) suggest that, for this organism, cell size would be positively correlated to temperature, although an opposite pattern was obtained for one strain (Kulk et al., 2012). In *Synechococcus*, previous results are also contradictory, Fu et al. (2007) measured the cellular quota of C in *Synechococcus* sp. WH7803 (CCMP1334) acclimated at 20°C or 24°C and unveiled a decrease of 32–34% at the highest temperature. However, in an analysis of three *Synechococcus*-like freshwater cyanobacterial isolates, no significant effects of temperature on cell size were found except for one strain, which displayed a larger mean cell volume with increasing temperature (Jezberová and Komárková, 2007).

A plausible reason for these contradictory results is the lack of consideration of the different cell-cycle stages within a population when analyzing the impact of temperature on their size. The cell cycle is a coordinated succession of events that encompass cell growth, DNA synthesis, DNA replication and end up with cell division (Liu et al., 2017). As cells progress through the division cycle, cell size increases until the moment of division, when cells have double their volume at birth (Forster et al., 2013). Thus, along with the expected direct effect of temperature on cell size (Atkinson, 1994; Atkinson et al., 2003), there is an indirect effect via changes in age-structure, that further complicates the study of temperature related size-patterns. Accordingly, in multicellular organisms, the relationship between temperature and size is typically evaluated separately for the different phases of the life cycle of the organism (i.e., larval, juvenile, adult stages) (Atkinson, 1994; Forster and Hirst, 2012). Yet, studies on unicellular organisms have generally obviated the existence of different growth stages with size differences in their life cycle (i.e., phases G1, S, G2/M of the division cycle). Due to its effect on the intrinsic growth rate of the population (Brown et al., 2004; Boyd et al., 2013), it is expected that temperature would modify the age structure of the population, i.e., the percentage of cells at each phase of the cell division cycle.

To our knowledge, the effect of temperature adaptation on the sizes of mother and daughter cells in an unicellular organism has been only analyzed in a single previous study (on the ciliate *Cyclidium glaucoma*) (Forster et al., 2013), which found that cell size of both age-classes displayed the negative relationship predicted by the TSR. These authors, however, did not relate changes in the temperature-cell size relationship with changes in the age-structure of the population. It should be considered that if the structure of the population in the different phases of the cell-division cycle changes with temperature, the evaluation of the temperature-size relationship for the average cell-sizes of the whole population would give spurious results.

Considering the synchrony in the cell division cycle of marine cyanobacteria following the photoperiod (Jacquet et al., 2001a, b), *Prochlorococcus* and *Synechococcus* are particularly suitable organisms for evaluating the effect of temperature on cell size at different cell-cycle stages. Here, we followed an experimental approach to test the applicability of the TSR to two ecologically relevant strains of marine cyanobacteria: *Prochlorococcus marinus* MIT9301 and *Synechococcus* sp. RS9907. We studied the effect of temperature on their growth rate, cell division cycle and the corresponding relationships between temperature and cell size, taking into account differences produced by changes in the age-structure of the populations.

## Materials and Methods

### Growth Conditions and Thermal Acclimation Process

*Prochlorococcus marinus* MIT9301 (RCC3377, hereafter MIT9301) and *Synechococcus* sp. RS9907 (RCC2382, hereafter RS9907) were obtained from the Roscoff Culture Collection (Roscoff, France). These two strains were selected as environmentally relevant as RS9907 is the strain that recruited the highest number of petB reads from the metagenomic Tara Oceans dataset (2009–2011) assigned to *Synechococcus*, and MIT9301 was one of the three strains that recruited the highest number of petB reads from the Tara Oceans dataset assigned to *Prochlorococcus* in the same dataset [as determined by Farrant et al. (2016)]. Both strains were grown in PCRS-11 Red Sea Salt based medium (Rippka et al., 2000) in non-axenic batch cultures. We modified the original recipe of PCRS-11 Red Sea Salt medium by adding 40 g salt L-1 (instead of the 33 g L-1 established in the original recipe) in order to obtain a salinity of 36, more representative of oceanic conditions (Antonov et al., 2010). Cultures were grown in polycarbonate flasks with vented caps under an irradiance of ca. 120 μmol quanta m-2s-1 with a 12:12 h photoperiod. Thermal acclimation of the cultures started from 22°C (temperature of maintenance at the Roscoff Culture Collection), and temperature was progressively changed by a maximum of 2°C at each acclimation step. As more extreme temperatures were approached, we reduced the temperature increase at each acclimation step down to 0.2°C in order to avoid lethal thermal stress. During the acclimation process and until the end of the experimental work, cultures were maintained in exponential growth phase by re-inoculation before cell density reached 30% of the maximum yield at each temperature as determined in preliminary analysis. Cultures were grown for a minimum of 8 generations at each acclimation step before changing the temperature. We considered that full acclimation to each treatment temperature had been reached when growth rates stayed stable for a minimum of at least two consecutive growth curves (a minimum of 8 generations), before starting the experiments. During the acclimation process and the experiments, the changes in cell abundance and size were monitoring by flow cytometry.

### Experimental Set-Up

For each of the two strains selected for this study, we performed the experimental work in two phases. In phase I, 160 mL replicate batch cultures (2 or 3 replicates) were acclimated to 19, 22, 25, and 30°C for MIT9301 and 20, 24, 26, 28, and 30°C for RS9907. Cultures were sampled daily 3 h after the initiation of the light period in order to characterize their growth response to temperature. In phase II, we analyzed the relationship between temperature and cell size during a 24 h cycle, thus taking into account the different cell-cycle phases. After initial re-inoculation, the growth of three biological replicates (50 mL) of each strain was followed at the different temperatures by sampling 3 h after the initiation of the light period. Once they reached the middle of the exponential phase, cultures were sampled every 2 h for 24 h in order to cover a full diel cycle. Phase II was carried out at the same temperatures as phase I, except the temperature of 22°C for MIT9301, due to a failure of the corresponding incubator chamber.

### Flow Cytometry

Samples for flow cytometric analysis were fixed with glutaraldehyde (final concentration of 0.025%) and incubated for 10 min at room temperature and in dark conditions, flash-frozen in liquid nitrogen and stored at −80°C. Samples from phase I experiments were thawed at room temperature and analyzed unstained, based on the natural fluorescence of the photosynthetic pigments of cyanobacterial cells, as described in Marie et al. (1999). Samples belonging to phase II were thawed and stained with Sybr Green II (Invitrogen, Thermo Fisher Scientific, Waltham, MA, United States; 10X final concentration) and incubated at room temperature for 10 min in the dark to perform cell size analysis during the diel cycle (Marie et al., 1997). Light fluorescence and scatter parameters were recorded using a FACSCalibur flow cytometer (Becton Dickinson; Franklin Lakes, NJ, United States) for *Prochlorococcus* samples and a FACSCanto II (Becton Dickinson) for *Synechococcus* samples (excitation wavelength: 488 nm for both equipment). Natural logarithmic-transformed side scatter (SSC) was used as a proxy of cell size and cell diameter (in μm) was estimated using the calibration provided in Calvo-Díaz and Morán (2006). We computed cell volume using cell diameter values, assuming a spherical shape for MIT9301 and RS9907 cells (Waterbury et al., 1979; Chisholm et al., 1988). Relative DNA content was inferred from green fluorescence (FL1, 530 μm) on stained samples and all flow cytometry parameters were normalized with respect to polystyrene spherical beads standards of 1 μm diameter (Molecular Probes, Thermo Fisher Scientific, Waltham, MA, United States). Cell cycle analysis used linear green fluorescence corresponding to the stain (FL1).

### Growth Rate and Division Cycle Analysis

Growth rate (μ, Day-1) were computed as the slope of a Ln (Nt) vs time plot, where Nt is the cell abundance at time t, and by considering only the samples taken during the exponential phase. For the analysis of the division cycle we used the ModFit LT™ software (Verity Software House; Topsham, ME, United States), which uses an algorithm for the deconvolution of the DNA histogram and fit two Gaussian curves for the G1 and G2 phases of the division cycle and a polygon for the S phase. Mean values of green fluorescence (FL1) for the S phase and the means and standard deviations of the G1 and G2 peaks were obtained, and 90% confidence intervals were calculated from the G1 and G2 means. These mean values and confidence intervals were subsequently used to delineate the populations of cells in each phase of the division cycle in cytograms analyzed using the packages flowViz (Ellis et al., 2019a) and flowCore (Ellis et al., 2019b) of the R statistical software (R Core Team, 2019). This approach allowed us to obtain abundances and SSC values for each phase of the division cycle (i.e., G1, S and G2).

### Exploring the TSR on a Discrete Growth Stage: New-Born Cells at G1 Phase

**FIGURE 1:**
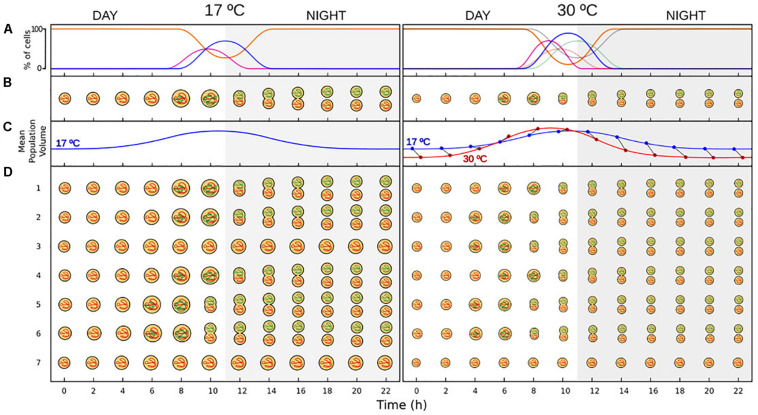
Schematic depiction of the effect of temperature on a marine cyanobacteria model population (based on previously published results and on this study). **(A)** Diel changes in the percentage of cells in each of the phases of the division cycle, in which cell cycle is synchronized with photoperiod. During the light period most of the population is at G1 phase of the division cycle (orange line), and replication (S phase – purple line) usually take place at late afternoon. Division started at dusk (17°C) or at the end of the afternoon (30°C), and a peak in the percentage of cells in G2 phase (blue line) is visible at dusk or at the first hours of the dark period (gray shaded area). At the middle of the dark period, division has finished in those cells that have accomplished a round of replication and a new cohort at G1 phase is added to the population. Note the difference in amplitude and timing of the phases across temperatures. Higher temperatures lead to a shortening of the length of the phases (Olson et al., 1986), as well as an increase in the percentage of cells that divide on a diel cycle (this study). Gray lines at 30°C represent the cell cycle at 17°C, presented here for comparison. **(B)** Changes in size for a cell that followed the division pattern described in A. Cell size increases during the first hours of the light period. Cell volume during division are double that of new-born G1 cells across generations of thermally acclimated populations (Forster et al., 2013). **(C)** Changes in the mean cell size of the population. Cell cycle is synchronized with photoperiod as showed in A, so most of the cells of the population followed the changes in cell size displayed in B. Blue line at 30°C represents the diel changes in cell size at 17°C, presented here for comparison. Notice the difference in amplitude and timing across temperatures, which causes SSC-trajectories to cross in a diel cycle, affecting the strength and direction of the relationship. Blue and red points represent the cell size at 17°C and 30°C, respectively, at each of the sampling times. **(D)** Age-structure of a phytoplankton population with cell cycle synchronize with photoperiod as described in A. Most of the population follow the pattern displayed in B (cells in rows 1, 2, and 4 at 17°C; and cells in rows 1, 4 at 30°C), while some cells progress faster through the division cycle (rows 5 and 6 at 17°C; rows 2 3, 5, 6 at 30°C), and other cells progress slowly and do not divide during the dark period (rows 3 and 7 at 17°C; and row 7 at 30°C). Cells that divide earlier start the day with a larger cell size (e.g., cell in row 5 at time 1 is equal in size to cell in row 1 at time 2).

In order to test the TSR on a discrete growth stage of the population, we developed an approach based on size-class histograms, which allowed us to select new-born cells at the G1 phase of the cell division cycle. Cell size of *Synechococcus* and *Prochlorococcus* increases during the first hours of light both in cultures and in natural populations, as a result of accumulation of new biomass derived from photosynthesis, and start to decrease at dusk when larger mother cells divide into new-born cells (Jacquet et al., 2001a; Zinser et al., 2009; Sato et al., 2015; Figure 1). Thus, we used those sampling times at dark when cells were decreasing in SSC as the result of the formation of new-born, smaller G1 cells resulting from cell division. For each of these sampling times, we computed size frequency distributions of G1 cells and subtracted the frequency distribution from the previous time. The resulting histogram reflected the size frequency distribution of the new-born cells. To consider that the new histogram adequately represents the size-distribution of new-born cells, abundance of G1 cells should increase from one sampling time to the next, as the culture is exponentially growing and a new cohort of G1 cells is added from one sampling time to another. We computed the median of the cell size distribution across sampling time points as a proxy of the cell size of new-born G1 cells for each replicate.

All statistical analysis were performed using R statistical software (R Core Team, 2019), analysis of variance tests and ordinary least square regression were calculated using standard functions in the R core package.

## Results

### Division Cycle, Diel Changes in Cell Size and Temperature

**FIGURE 2:**
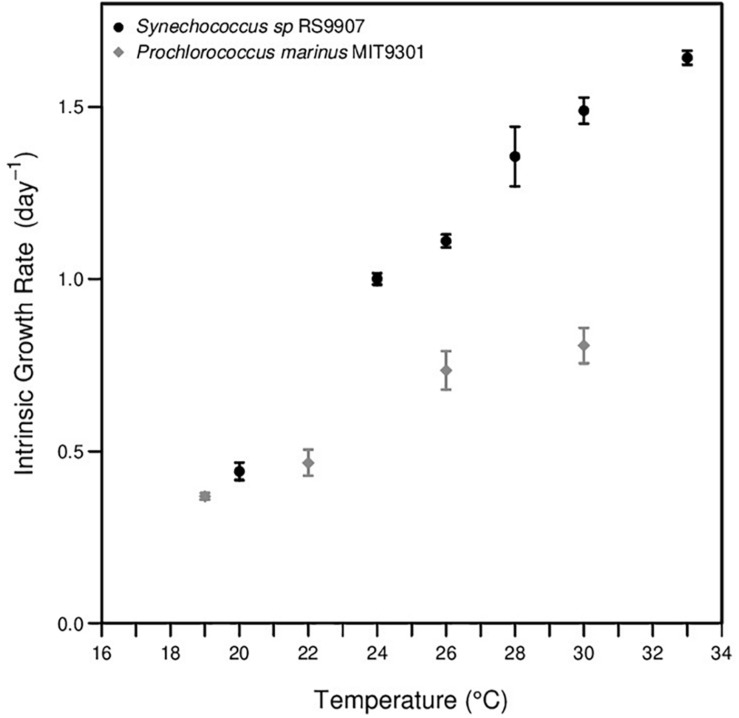
Growth response to temperature of *Prochlorococcus marinus* MIT9301 (gray points) and *Synechococcus* sp. RS9907 (black points).

In phase I experiments, the intrinsic growth rates of MIT9301 and RS9907 were obtained after long-term acclimation as explained in the “Materials and Methods” section. The growth rate of MIT9301 enhance from ca. 0.37 at 19°C to 0.73 at 26°C, and then it slightly increased up to ca. 0.8 day-1 at 30°C (Supplementary Table S1, Figure 2). Similarly, growth rates of RS9907 increased linearly from ca. 0.44 day-1 at 20°C to ca. 1.34 day-1 at 28°C, and above that temperature growth rate continued increasing but at lower pace, displaying values of ca. 1.43 and 1.58 day-1 at 30 and 33°C, respectively (Figure 2).

**FIGURE 3:**
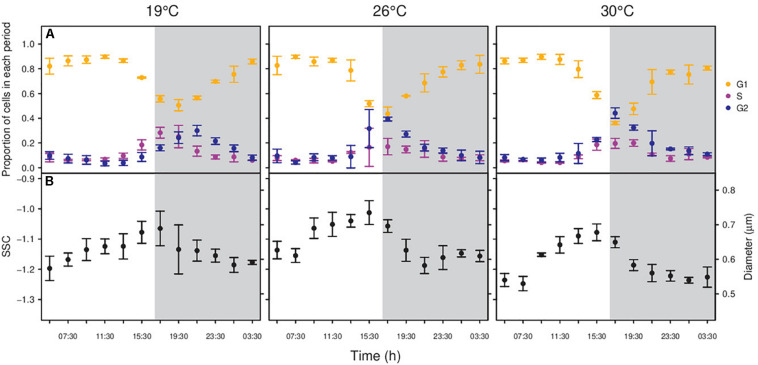
Changes in the proportion of cells in each period of the division cycle along a diel cycle for *Prochlorococcus marinus* MIT9301 **(A)** and diel periodicity in SSC for the whole population **(B)**. Gray shaded area represents the dark period. Black points – All MIT9301 cells, regardless the period of the division cycle. Orange points – cells in G1 phase of the division cycle. Purple points – cells in S phase. Blue points – cells in G2 phase. Error bars denotes s.d. (standard deviation).

**FIGURE 4:**
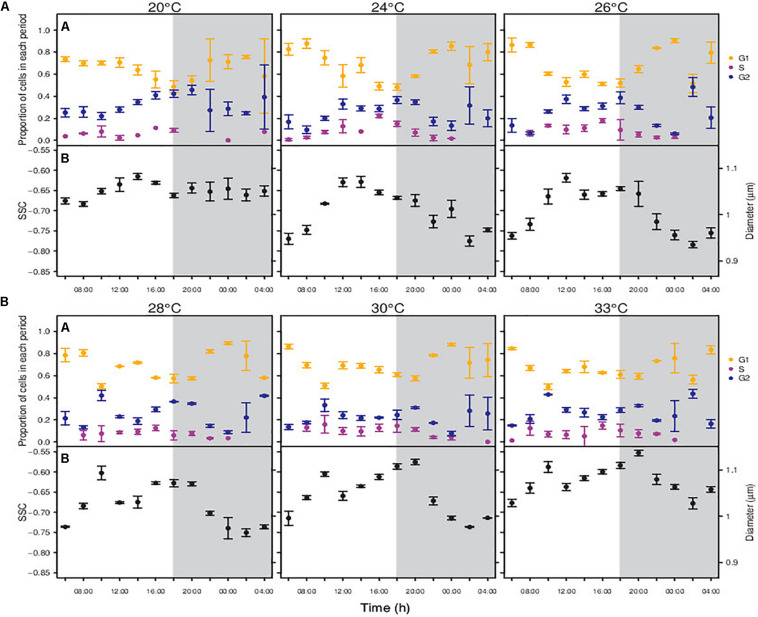
Changes in the proportion of cells in each period of the division cycle along a diel cycle for *Synechococcus* sp. RS9907 **(A)** and diel periodicity in SSC for the whole population **(B)**. Gray shaded area represents the dark period. Black points – All RS9907 cells, regardless the period of the division cycle. Orange points – cells in G1 phase of the division cycle. Purple points – cells in S phase. Blue points – cells in G2 phase. Error bars denotes s.d.

In phase II experiments, changes in the proportion of cells in each phase of the division cycle were observed during the 24 h (Figures 3A, 4A). As expected, cells increased in size as they went forward in the division cycle, with the mean cell diameter increasing from G1 < S < G2 phases for both strains (all *t*-test *p*-values <0.01; Figures 3, 4). During the light hours, MIT9301 population remained mainly in G1. Just before midday, cells began the synthesis of DNA, with the proportion of cells in S phase peaking at the transition from light to dark. Following the increase in the proportion of cells in the S phase, there was an increase in the number of cells in G2 as cell division progressed. Cytokinesis (division of the cytoplasm in two daughter cells) took place during the dark period, and at dawn most cells were in the G1 phase again. Due to this pattern of division, the abundance of MIT9301 cells remained fairly constant during the light hours and increased mostly during the night (Supplementary Figure S1). Temperature affected the division cycle of MIT9301 in two ways. First, higher temperatures brought forward the onset of the DNA replication (Figure 3A). Cells grown at 26°C and 30°C entered G2 4 h earlier (all six replicates at 17:30) than cells grown at 19°C (all three replicates 21:30). Second, temperature affected the proportion of cells that enter a new round of replication (ANOVA, *p*-value < 0.01; Supplementary Figure S2), with the proportion of cells that entered G2 increasing with temperature (25.29, 38.26, and 42.50% at 19, 26, and 30°C, respectively).

The division pattern of RS9907 was different to that displayed by MIT9301. At temperatures from 24 to 33°C, RS9907 cells showed ultradian growth (sensu Shalapyonok et al., 1998), based on G2 phase an not S phase), i.e., they went through more than one round of division along the daily cycle. Indeed, the proportion of cells in G2 phase peaked three times during the 24-h cycle, with one peak around midday, another at dusk and again a slightly increase at the end of the dark period (Figure 4A). Consequently, cell abundance increased during the light and also during the dark period (Supplementary Figure S3). At the lowest extreme of the temperature gradient (20°C), RS9907 showed a single round of division. Cells entered progressively the synthesis phase during the light hours, with a peak in the proportion of cells in G2 at the transition from light to dark, and cytokinesis took place during the dark, similarly to MIT9301.

In connection with the synchrony of the cell cycle with the photoperiod, cells from both strains displayed temperature-dependent diel changes in SSC. MIT9301 cells showed one maximum and one minimum SSC value per day (Figure 3B). Cell size was maximum at the beginning of the dark period (19°C), or just before the dusk (26 and 30°C); and it decreased until its minimum value before dawn (19 and 30°C) or around the middle of the dark period (26°C). Temperature affected the amplitude of the daily oscillations in cell size: cell volume increased by 73.87, 101.39, and 109.50% at 19, 26, and 30°C, respectively.

Cells of RS9907 displayed one or two daily SSC maximum and minimum, depending on the experimental temperature (Figure 4B). At the lowest temperatures of 20 and 24°C, SSC peaked once per day with cell size increasing during the beginning of the light hours and showing maximum values around midday. Then, SSC decreased until it displayed the smaller size at the dark period. At temperatures of 26, 28, 30 and 33°C, SSC peaked twice per day: cell size started to increase with the initiation of the light period until a maximum around midday, then slightly decreased, and increased again during the last hours of the light period until showing a new maximum at the beginning of the dark period. After that, cell size decreased reaching a minimum 4 h before the next light period.

### Division Cycle and the Temperature-Cell Size Relationship

**FIGURE 5:**
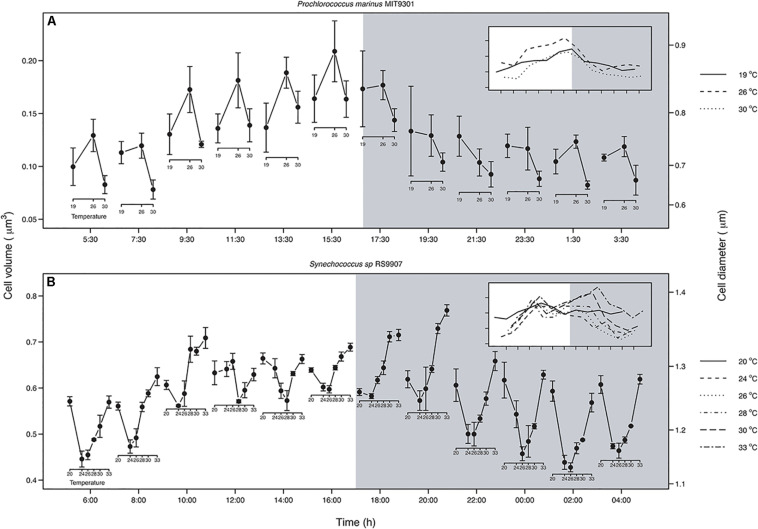
Relationship between temperature and cell volume along a diel cycle for *Prochlorococcus marinus* MIT9301 **(A)** and *Synechococcus* sp. RS9907 **(B)**. White and gray backgrounds correspond to the light and dark periods, respectively. The insets show the SSC patterns for each temperature; note that when the curves are crossing each other, there is a change in the shape of the relationship. MIT9301: solid line – 19°C; dashed – 26°C; dotted – 30°C. RS9917: solid line – 20°C; dashed – 24°C; dotted – 26°C; dotdash – 28°C; longdash – 30°C; twodash – 33°C. Error bars denotes s.d.

When considering the whole population, the relationship between temperature and cell size varied along the day for both strains (Figure 5). In MIT9301, the shape of the relationship did not change along the light hours and showed maximum cell size at 26°C. In contrast, at the beginning of the dark period, cell size at 19°C started to increase relative to the one at 26°C, and by the middle of the dark period the relationship became negative, as proposed by the TSR (Figure 5A). At the end of the dark period, cell size at 19°C decreased again, and hence the relationship between temperature and cell size recovered the convex shape found during the light hours.

For RS9907 the pattern was different from that found in MIT9301. At the beginning of the light hours the relationship was concave: cell size decreased from 20 to 24°C, and then increased linearly until 33°C where it reached a cell size similar to that at 20°C (Figure 5B). During the first hours of the light period the concave pattern was maintained, but with slight differences in the cell volumes at temperatures located in the middle of the thermal tolerance curve. At the end of the light period, size differences between temperatures located at the edges of the thermal tolerance curve and those located at the middle decreased, thus cell size at 30 and 33°C was greater than that displayed at 19°C. When cells entered the dark period, cell size at 20°C decreased until a value similar to that at 24°C, and hence the reversal of the TSR was displayed. This reversal pattern was confined to the first 2 h of the dark period, and by the middle of the night the relationship recovered the concave shape. When considering the average size of the whole population over the entire temperature range, RS9907 did not display the predicted negative response of cell size to increasing temperature at any point of the diel cycle (Figure 5B).

### Temperature and Cell Size of New-Born Cells

**FIGURE 6:**
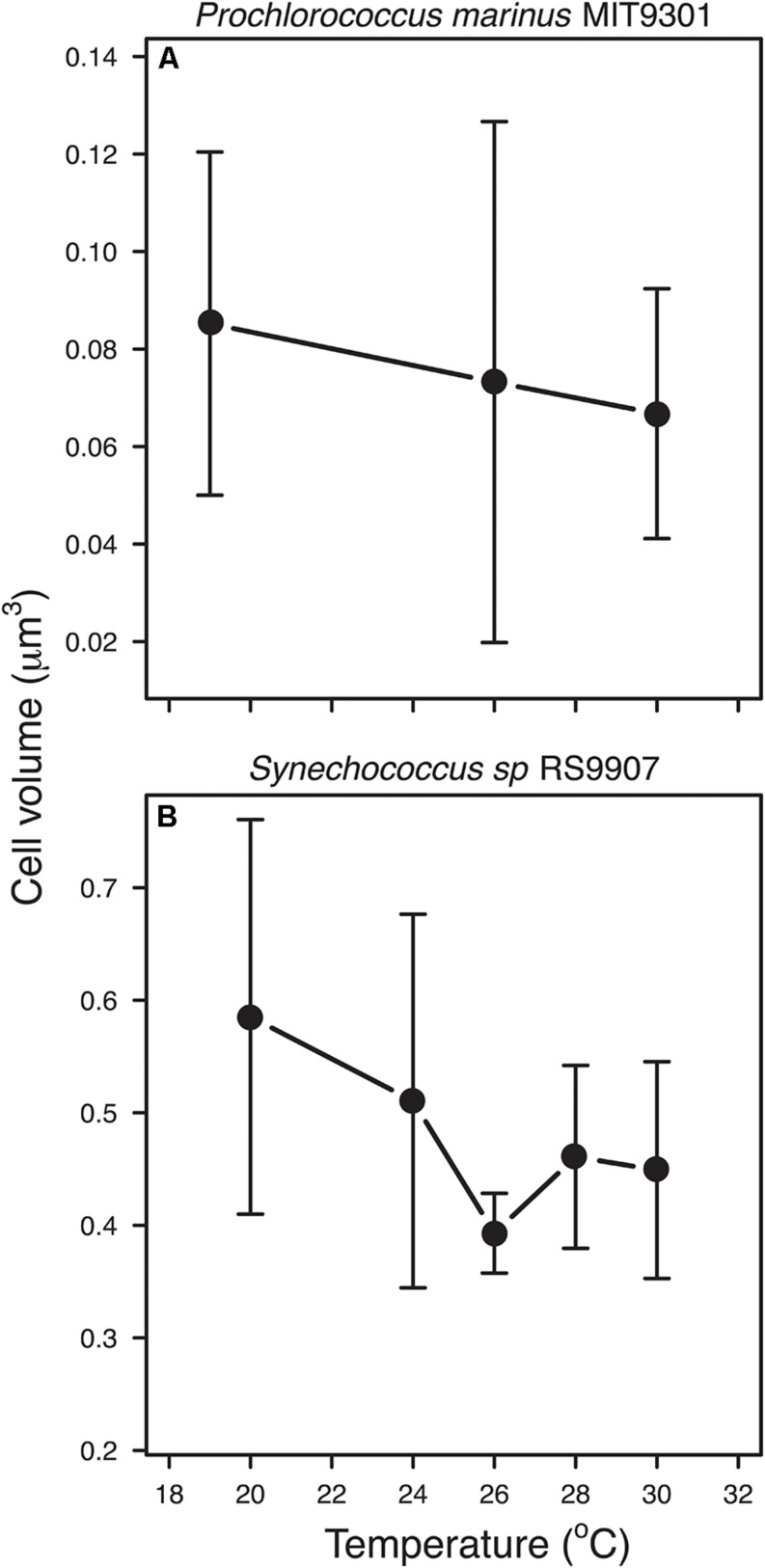
Relationship between temperature and average cell volume of new-born cells for *Prochlorococcus marinus* MIT9301 **(A)**, and *Synechococcus* sp. RS9907 **(B)**. Error bars denotes s.d.

Median cell volume of new-born cells (see section “Materials and Methods”) followed the negative trend predicted by the TSR in both strains, with increasing temperatures leading to smaller cell sizes. However, the slope of the regression was not significant for MIT9301, probably due to the scarcity of data points (analysis of covariance test: RS9907: *n* = 34, F1,34 = 4.373, *p* = 0.044; MIT9301: *n* = 28, F1,26 = 1.008, *p* = 0.325; Figure 6). In order to compare our results with those obtained by Atkinson et al. (2003), we followed their procedure and scaled the cell volumes of new-born cells to the predicted cell volume at 15°C (V15) for each species, i.e., for each cell volume value, we calculated the difference with V15 and then divide it by V15. Regression of pooled data showed that mean thermal sensitivity (±s.e.) of cell volume (scaled at 15°C) was −0.022°C-1 (±0.011; F1,62 = 4.169, *p* = 0.045).

## Discussion

One of the main conclusions of this work is that testing the TSR in unicellular organisms is more challenging than previously considered in earlier studies. Different results can be reached depending on whether the whole population is analyzed, or different cell-cycle stages are considered. We did not find support for the expected negative linear relationship between temperature and average population cell size for any of the two strains analyzed (Figure 5). Instead, we unveiled changes in the direction and strength of the relationship between temperature and cell size on a daily scale for both strains. This dynamic relationship arises as a consequence of the differences in the phasing and amplitude of diel oscillations in cell size across temperatures, which forces daily SSC trajectories to cross. For example, differences in the timing of minimum SSC for MIT9301 among temperatures of 19 and 26°C change the direction of the relationship between temperature and cell size from positive during the light hours to negative, as predicted by the TSR, at the middle of the dark period (Figure 4A). The variability observed in the relationship between temperature and average cell size for the same strains along the diel cycle, presumably in response to changes in population age-structure, may reconcile previous inconsistent results when testing the TSR on unicellular phytoplanktonic cells.

A more appropriate approach to test the TSR should consider the effect of temperature on cell size at specific cell-cycle stages instead of whole-population averages. The estimation of the size of new-born cells allowed us to test the prediction of the TSR for a discrete cell cycle stage of the population, removing the confounding factors associated with the differences in the age structure across experimental temperatures, and temporal differences associated with the timing in the division cycle. Based on this approach, the negative relationship proposed by the TSR was confirmed for both strains, supporting the view of the universality of this rule among different types of organisms (Atkinson, 1994; Atkinson et al., 2003). Remarkably, our values of thermal sensitivity of cell size for the combined dataset of MIT9301 and RS9907 (−0.022°C-1 ± 0.011 s.e.) also agree with the previous estimate reported by Atkinson et al. (2003) in their meta-analysis in unicellular organisms (−0.025°C-1 ± 0.004 s.e). Given that during normal growth one mother cell is divided into two daughter newborn cells (Liu et al., 2017) the same temperature dependent pattern could be expected for adult, mother cells although we could not find a way to test the TSR for other cell-cycle states.

In most phytoplankton species, the cell cycle is coupled with the photoperiod and the burst of replication and division is restricted to a discrete temporal window in the light:dark cycle (Vaulot et al., 1995; Jacquet et al., 2001a; Figure 1A). For *Prochlorococcus*, replication usually starts just before dusk or at the transition from light to dark, with cytokinesis occurring during the first hours of the dark period (Jacquet et al., 2001a, b; Zinser et al., 2009; Kolowrat et al., 2010). In contrast to *Prochlorococcus*, the cell cycle in *Synechococcus* exhibits a greater variability in timing with respect to the photoperiod, with replication taking place during the afternoon, at the transition from light to dark, or during the dark period depending on the strain [reviewed by Jacquet et al. (2001a)]. External factors can influence the progress and the duration of the different periods of the division cycle (Mella-Flores et al., 2012). However, despite the key role of temperature in regulating the metabolism of phytoplankton, its effects on the cell cycle have been seldom analyzed. To our knowledge, just a single early study has analyzed the effect of temperature on the division cycle of phytoplankton (the diatom *Thalassiosira weissflogii*, the prymnesiophyte *Pleurochrysis carterae*, and the dinoflagellate *Amphidinium carterae*; (Olson et al., 1986). The results from this work unveiled that suboptimal temperatures caused an elongation of all periods of the division cycle in similar proportions in these eukaryotic species.

In the case of MIT9301, the increase in the percentage of cells entering a new round of replication with temperature agrees with the positive relationship between temperature and growth rate, as more cells entering in the division cycle per round would imply higher growth rates. Drawing conclusions about the effect of temperature on the cell cycle of RS9907 is more challenging due to some ultradian growth observed in these cells at most temperatures, which caused proportions of G2 and to a lower extent S cells to peak several times during a diel cycle. Similarly, cell cycle distribution has been shown to be quite variable when comparing different *Synechococcus* strains (Binder and Chisholm, 1995). However, we still can report some generalities about the cell cycle in the strain RS9907. First, the increase in abundance and in the percentage of cells in G1 during the dark hours, linked with the decrease in the percentage in G2 cells, suggests that the light-restriction point at G2 in this strain is absent or weaker than in other *Synechococcus* strains (Jacquet et al., 2001a). In fact, this absence of restriction point, i.e., cell cycle arrest at specific blocking points in the cell cycle, allowed us to select new-born cells at dusk and test the TSR on a single discrete stage (new-born cells at G1 with no growth in size). Second, the presence of more than one peak in the percentage of cells in G2 phase suggests the existence of several periods of division in a single day, evidenced as much higher intrinsic growth rates compared with MIT9301 (Figure 2).

The lack of similarity between previous work on the timing of *Synechococcus* cell cycle and our results could be due to a strain-specific response, but it could also arise as result of the short generation times displayed at most of the experimental temperatures. Although previous studies have shown generation times shorter than 1 day, our estimates of generation times are even shorter than those typically reported in studies measuring the timing of replication under light:dark cycles (Campbell and Carpenter, 1986; Sweeney and Borgese, 1989; Jacquet et al., 2001a). As an example, doubling or generation time at 30°C was ca. 11 h, which means that the complete population should go through two rounds of division in a single day, with at least a small fraction of the population completing a third round of division. Despite these short generation times, the DNA histograms showed a bimodal frequency distribution, suggesting that a new round of replication did not start until cytokinesis was completed (Binder and Chisholm, 1990; Burbage and Binder, 2007), in accordance with the slow-growth mode proposed by Helmstetter and Cooper (1968).

Regarding the daily variation in cell size, our results with MIT9301 show a similar pattern to previous studies on *Prochlorococcus* strains: cells increase in size during the light hours, peaking at the transition from light to dusk, and then cell size starts to decrease, displaying minimum values around dawn. This pattern was similar at all temperatures in MIT9301. Contrary, in the case of RS9907, we found differences in the diel pattern of cell size among temperatures. This difference is because at temperatures displaying ultradian growth, cell size peaked twice in a day, while cell size at 20°C peaked only once. Proposed mechanisms for the daily variation in cell size are the accumulation of biomass derived from photosynthesis during the light hours and the division of cells and respiration during the dark hours (Jacquet et al., 2001a; Hunter-Cevera et al., 2014; Sato et al., 2015).

Altogether, this study highlights the importance of considering the age structure of the populations when exploring the temperature-cell size relationship in unicellular organisms, as has been typically done on multicellular organisms. We have showed how differences in the age-structure of the populations obscure the true nature of the relationship between temperature and cell size, i.e., the negative relationship emerges when evaluated on a single stage, and this could be one of the reasons that contribute to the different responses described in unicells (Atkinson et al., 2003). Studies testing the TSR on multicellular organisms have considered the existence of different growth or development stages in the life cycle of these organisms (Atkinson, 1994; Forster and Hirst, 2012). For example, identification of different stages among the larval period in the crustacean *Artemia franciscana* unveiled a change in the direction of the TSR during ontogeny: from an inverse TSR during early larval stages to the predicted TSR at the end of the larval stage (Forster and Hirst, 2012). If the relationship among temperature and size were not evaluated independently for the different development stages, a single pattern in the relationship would have been wrongly assigned to the larval stage.

We conclude that studies exploring the TSR on unicellular organisms should bear in mind the differences in the age-structure of the populations, as studies on multicellular organism do. In this study we presented a method which combining flow cytometry, cell cycle analysis and size frequency distributions allows for the identification of cells on a particular growth stage of the cell cycle, but methods based on visual identification of cells could also be applied for the same purpose.

## Data Availability Statement

The datasets generated for this study are available on request to the corresponding author.

## Author Contributions

AP, AC, AL, and FG carried out the experimental work and collected data. LA-S and ÁL-U designed the study. AP wrote the first draft of the manuscript, and all authors contributed substantially to revisions.

## Conflict of Interest

The authors declare that the research was conducted in the absence of any commercial or financial relationships that could be construed as a potential conflict of interest.
