# Supplementary material for: Changes in Population Age-Structure Obscure the Temperature-Size Rule in Marine Cyanobacteria
Source: Front Microbiol. 2020 Aug 28;11:2059. doi: 10.3389/fmicb.2020.02059 (PMC7485217; doi:10.3389/fmicb.2020.02059)
Supplement: Supplementary file 1 [file Table_1.docx]

Supplementary Information


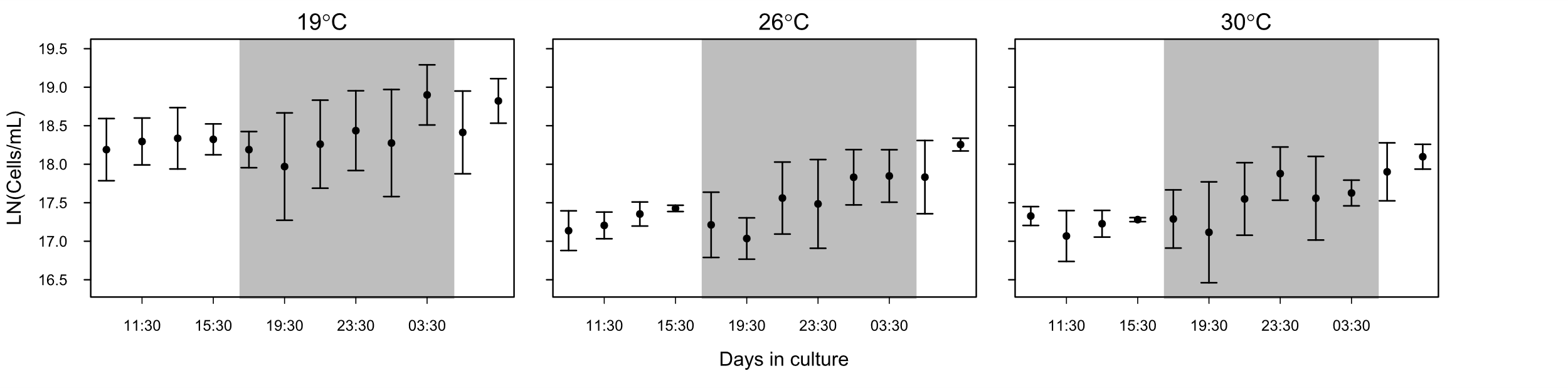


**Figure S.1.** Abundances of *Prochlorococcus marinus* MIT9301 along the diel cycle (*Phase II* – Materials and Methods). The last two sample times presented here (05:30 and 07:30 02/15/2018) were rearranged to the beginning of the cycle in Figures 4 and 5 (as 05:30 and 07:30 02/14/2018) for clarity on visualization of diel changes in cell size and cell cycle properties.


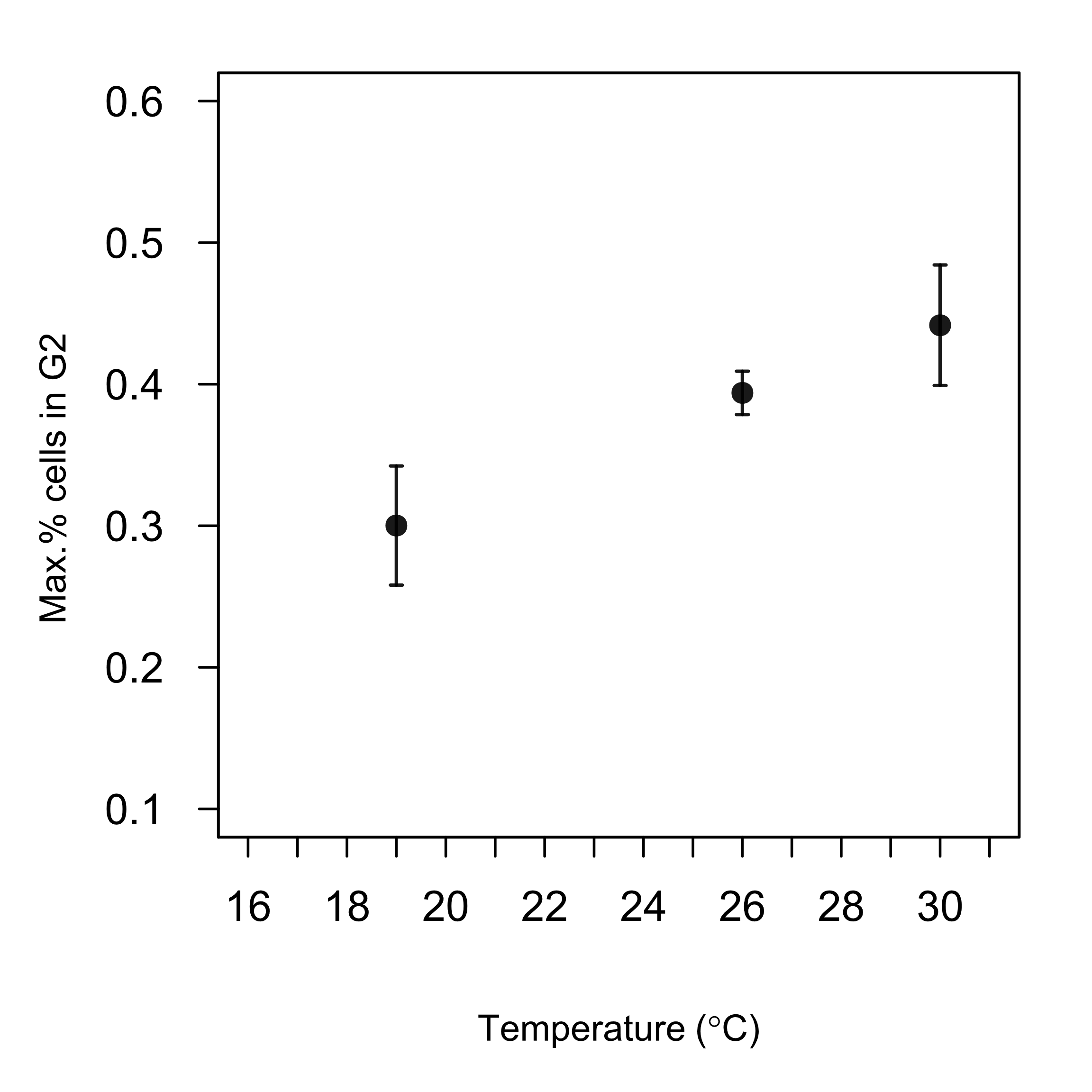


**Figure S.2.** Relationship between temperature and the maximum percentage of cells that enter in a new round of replication (G2 phase) in a diel cycle for *Prochlorococcus marinus* MIT9301.


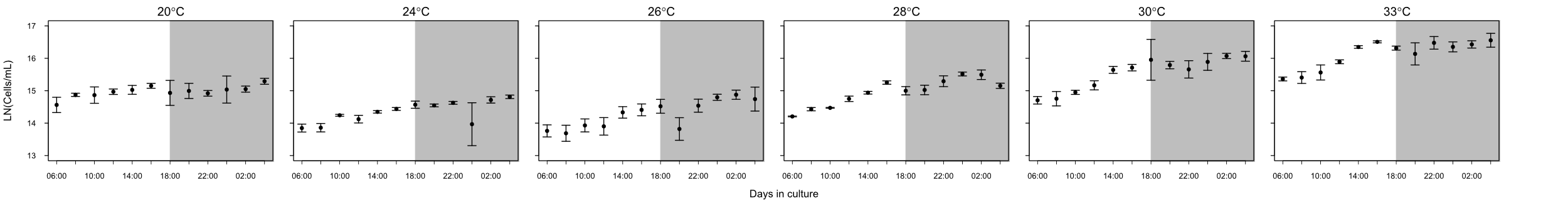


**Figure S.3.** Abundances of *Synechococcus sp.* RS9907 along the diel cycle (*Phase II* – Materials and Methods).

**Table S.1.** Growth rates of *Prochlorococcus marinus* MIT9301 and *Synechococcus sp.* RS9907.

| **Strain** | **Temperature (**°C**)** | **Replicate** | **Growth rate (day^-1^)** | **s.e.** | **p-value** | **r.squared** | **n^1^** |
| --- | --- | --- | --- | --- | --- | --- | --- |
| *Prochlorococcus marinus* MIT9301 | 19 | 1 | 0.379 | 0.029 | 0.006 | 0.989 | 4 |
|  | 19 | 2 | 0.368 | 0.108 | 0.077 | 0.853 | 4 |
|  | 19 | 3 | 0.360 | 0.074 | 0.039 | 0.923 | 4 |
|  | 22 | 1 | 0.439 | 0.023 | 0.000 | 0.986 | 7 |
|  | 22 | 2 | 0.450 | 0.036 | 0.000 | 0.969 | 7 |
|  | 22 | 3 | 0.510 | 0.041 | 0.000 | 0.969 | 7 |
|  | 26 | 1 | 0.671 | 0.092 | 0.018 | 0.964 | 4 |
|  | 26 | 2 | 0.763 | 0.062 | 0.007 | 0.987 | 4 |
|  | 26 | 3 | 0.771 | 0.121 | 0.024 | 0.953 | 4 |
|  | 30 | 1 | 0.751 | 0.119 | 0.024 | 0.952 | 4 |
|  | 30 | 2 | 0.818 | 0.105 | 0.016 | 0.968 | 4 |
|  | 30 | 3 | 0.851 | 0.109 | 0.016 | 0.968 | 4 |
|  |  |  |  |  |  |  |  |
| *Synechococcus* *sp*. RS9907 | 20 | 1 | 0.406 | 0.010 | 0.000 | 0.997 | 7 |
|  | 20 | 2 | 0.453 | 0.016 | 0.000 | 0.994 | 7 |
|  | 20 | 3 | 0.454 | 0.015 | 0.000 | 0.995 | 7 |
|  | 20 | 4 | 0.478 | 0.018 | 0.000 | 0.993 | 7 |
|  | 20 | 5 | 0.425 | 0.018 | 0.000 | 0.991 | 7 |
|  | 20 | 6 | 0.430 | 0.004 | 0.000 | 1.000 | 7 |
|  | 24 | 1 | 0.987 | 0.037 | 0.000 | 0.996 | 5 |
|  | 24 | 2 | 1.000 | 0.029 | 0.000 | 0.997 | 5 |
|  | 24 | 3 | 1.019 | 0.023 | 0.000 | 0.998 | 5 |
|  | 24 | 4 | 1.022 | 0.019 | 0.000 | 0.999 | 5 |
|  | 24 | 5 | 0.985 | 0.023 | 0.000 | 0.998 | 5 |
|  | 24 | 6 | 0.989 | 0.012 | 0.000 | 1.000 | 5 |
|  | 26 | 1 | 1.097 | 0.060 | 0.000 | 0.988 | 6 |
|  | 26 | 2 | 1.124 | 0.064 | 0.000 | 0.987 | 6 |
|  | 28 | 1 | 1.384 | 0.030 | 0.000 | 0.999 | 4 |
|  | 28 | 2 | 1.348 | 0.029 | 0.000 | 0.999 | 4 |
|  | 28 | 3 | 1.186 | 0.124 | 0.011 | 0.979 | 4 |
|  | 28 | 4 | 1.405 | 0.030 | 0.000 | 0.999 | 4 |
|  | 28 | 5 | 1.406 | 0.038 | 0.001 | 0.999 | 4 |
|  | 28 | 6 | 1.408 | 0.045 | 0.001 | 0.998 | 4 |
|  | 30 | 1 | 1.516 | 0.048 | 0.001 | 0.998 | 4 |
|  | 30 | 2 | 1.462 | 0.066 | 0.002 | 0.996 | 4 |
|  | 33 | 1 | 1.612 | 0.042 | 0.000 | 0.998 | 5 |
|  | 33 | 2 | 1.629 | 0.027 | 0.000 | 0.999 | 5 |
|  | 33 | 3 | 1.642 | 0.045 | 0.000 | 0.998 | 5 |
|  | 33 | 4 | 1.648 | 0.023 | 0.000 | 0.999 | 5 |
|  | 33 | 5 | 1.663 | 0.037 | 0.000 | 0.999 | 5 |
|  | 33 | 6 | 1.664 | 0.031 | 0.000 | 0.999 | 5 |

1. number of data points.
